# Clinical evaluation of the peripheral perfusion index as a predictor of femoral nerve block efficacy in unilateral arthroscopic meniscal surgery

**DOI:** 10.3389/fneur.2026.1562770

**Published:** 2026-05-26

**Authors:** Yao Gong, Qi He, Li Zhang, Wei Wei, Cuo Mao Ji Zhang

**Affiliations:** Affiliated Sport Hospital of Chengdu Sport University, Chengdu, Sichuan, China

**Keywords:** femoral nerve block, regional anesthesia, peripheral perfusion index, meniscal injury, knee arthroscopy, ultrasound

## Abstract

**Objective:**

To investigate the potential of Perfusion index (PI) as a reliable indicator for assessing the efficacy of femoral nerve block (FNB).

**Method:**

A total of 78 patients with knee meniscus injuries, admitted to Affiliated Sport Hospital of CDSU between June 2023 to March 2024, were recruited for this study. All patients had an American society of anesthesiologists (ASA) physical status classification of I or II and were scheduled to undergo unilateral arthroscopic meniscus surgery under general anesthesia supplemented with FNB. Patients who met the inclusion criteria were administered an ultrasound-guided FNB using 20 mL of 0.25% ropivacaine. Following the completion of the nerve block, the PI values of the big toe were monitored on both the blocked and non-blocked sides at two-minute intervals for a total duration of 20 min. Concurrently, the sensory and motor functions of the blocked limb were assessed at corresponding time points.

**Result:**

The area under the ROC curve (AUROC) of the PI value at T6 on the blocked side was 0.993 (95% CI: 0.982–1.000), with 96.7% sensitivity, 93.3% specificity, a Youden index of 0.934, and a cut-off value of 1.755. PI values differed significantly between the blocked and non-blocked sides at all time points (*p* < 0.001). On the blocked side, PI values varied significantly across time points (*p* < 0.001), with the highest increase at T6 (mean = 2.83 ± 0.72, 2.16 times the baseline). No significant changes occurred on the non-blocked side (*p* > 0.05). On the blocked side, male PI values increased more than female values (*p* < 0.001), while no gender differences were observed on the non-blocked side (*p* > 0.05).

**Conclusion:**

The PI represents a sensitive and straightforward method that can provide an effective and objective foundation for assessing the success of early FNB. A value above 1.755 indicates successful block, while little change suggests failure. This enables anesthesiologists to promptly adjust anesthetic protocols, enhance the efficiency of knee arthroscopic procedures, and align with the principles of Enhanced recovery after surgery (ERAS).

**Trial registration:**

This study was approved by the Ethics Committee of the Affiliated Sport Hospital of CDSU in December 2022 [Approval No: Affiliated Sport Hospital of CDSU Medical Ethics (2022) 45)] and subsequently registered with the Chinese Clinical Trial Registry in May 2023 (Registration No: ChiCTR2300071976).

**Clinical trial registration:**

www.chictr.org.cn, Registration number: ChiCTR2300071976.

## Introduction

Meniscus injury of the knee joint refers to damage to the meniscus caused by trauma, degenerative conditions, inflammatory diseases, and other etiological factors (1). Arthroscopic minimally invasive surgery is a commonly employed clinical treatment for this condition (2). In recent years, with the increasing application of ultrasound technology in clinical settings, this surgical approach is often combined with ultrasound-guided femoral nerve block (FNB), which not only reduces the requirement for anesthetic agents but also enhances patient comfort. However, optimizing the evaluation methodology for nerve block remains an urgent issue that requires further investigation. The efficacy of peripheral nerve block is primarily assessed based on patients’ motor and sensory changes. This conventional approach is subjective, time-consuming, and contingent upon patient cooperation (3). In pediatric patients, those with comorbid psychiatric conditions, and individuals with severe fractures, traditional evaluation methods may not be feasible. With advancements in ECG monitoring technology, several researchers have suggested that changes in Perfusion index (PI) could serve as an indicator for evaluating the effectiveness of nerve blocks (4).

The PI, derived from the Photoplethysmography (PPG) signal and measured by a percutaneous pulse oximetry monitor, is a noninvasive tool that is inexpensive, fast, and easy to use. It represents the ratio of light absorption at 940 nm between the pulsatile component AC (mainly pulsatile arterioles) and the non-pulsatile component DC (venous, capillary and non-pulsatile arterial blood and tissue) of the local tissue, namely AC940/DC940 (5). Peripheral nerve block blocks not only motor and sensory nerves but also sympathetic nerves, and sympathetic block precedes sensory nerve block. PI assesses the effect of block by sensing local hemodynamic changes triggered by sympathetic nerve activity (6).

This method has demonstrated satisfactory outcomes in brachial plexus block, sciatic nerve block, intraspinal anesthesia, and stellate ganglion block. However, there is currently a paucity of studies evaluating its efficacy for FNB. Therefore, we conducted this study with the aim of exploring whether PI can be used to evaluate the effect of FNB, and to provide a theoretical basis for optimizing the surgical procedure of knee arthroscopy.

## Materials and methods

### Patients

A total of 78 patients diagnosed with knee meniscus injuries were recruited from our hospital between June 2023 to March 2024. These patients were scheduled to undergo unilateral arthroscopic meniscectomy under general anesthesia supplemented with FNB. All patients met the following inclusion criteria: (1) ASA I-II classification, aged 18–65 years, with a body mass index (BMI) of 18–30 kg/m^2^; (2) underwent unilateral meniscal arthroscopy; (3) were able to comprehend and sign the informed consent form. Exclusion criteria included: (1) BMI ≥ 30 kg/m^2^; (2) history of peripheral vascular disease prior to surgery; (3) history of progressive neuromuscular disease preoperatively; (4) preoperative history of hypertension or diabetes; (5) preoperative neurological deficits; (6) preoperative mental disorders, language impairments, or inability to cooperate; (7) contraindications to local anesthesia, such as allergy to anesthetics, coagulation disorders, or local infection; (8) preoperative use of vasoconstrictor medications, including *α* or *β* blockers. Prior to initiating the trial, written informed consent was obtained from all participants and/or their legal guardians.

### Methods of anesthesia

**Figure 1:**
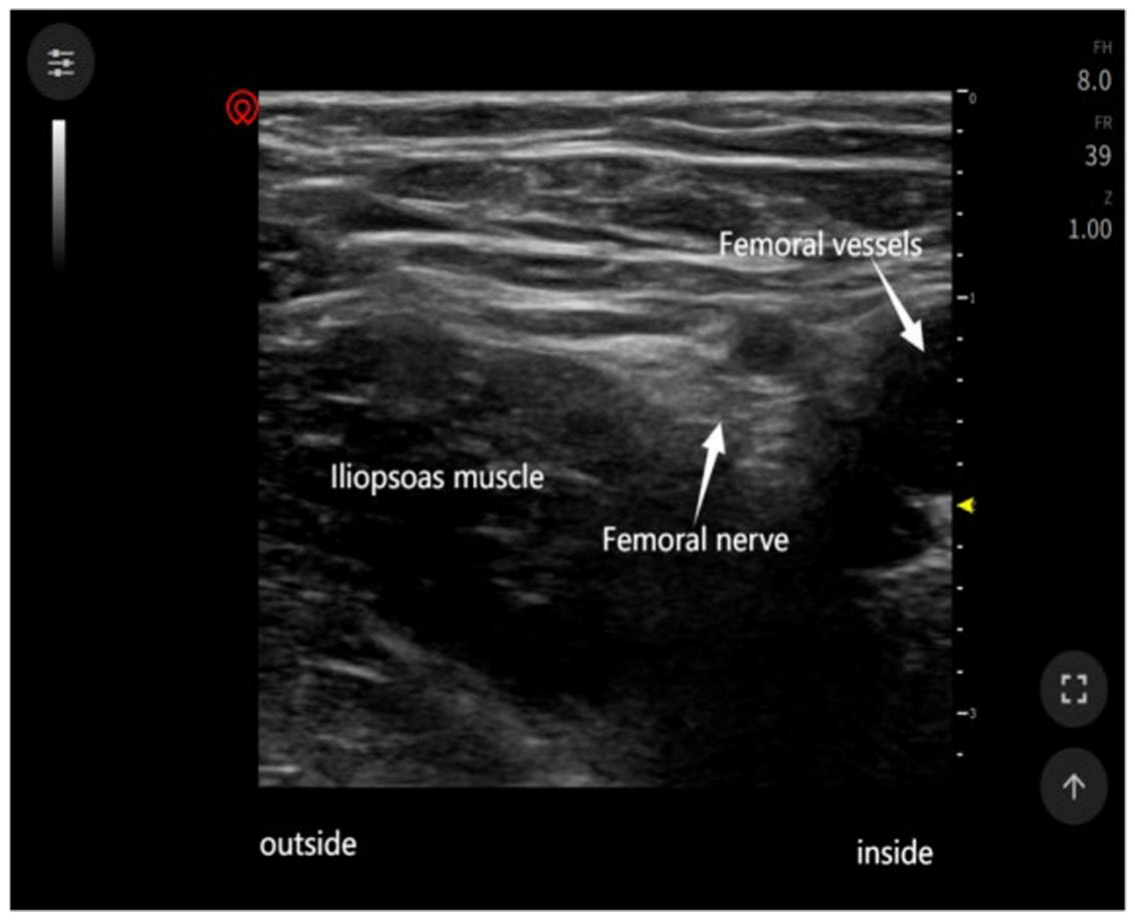
Ultrasound imaging of the FN.

**Figure 2:**
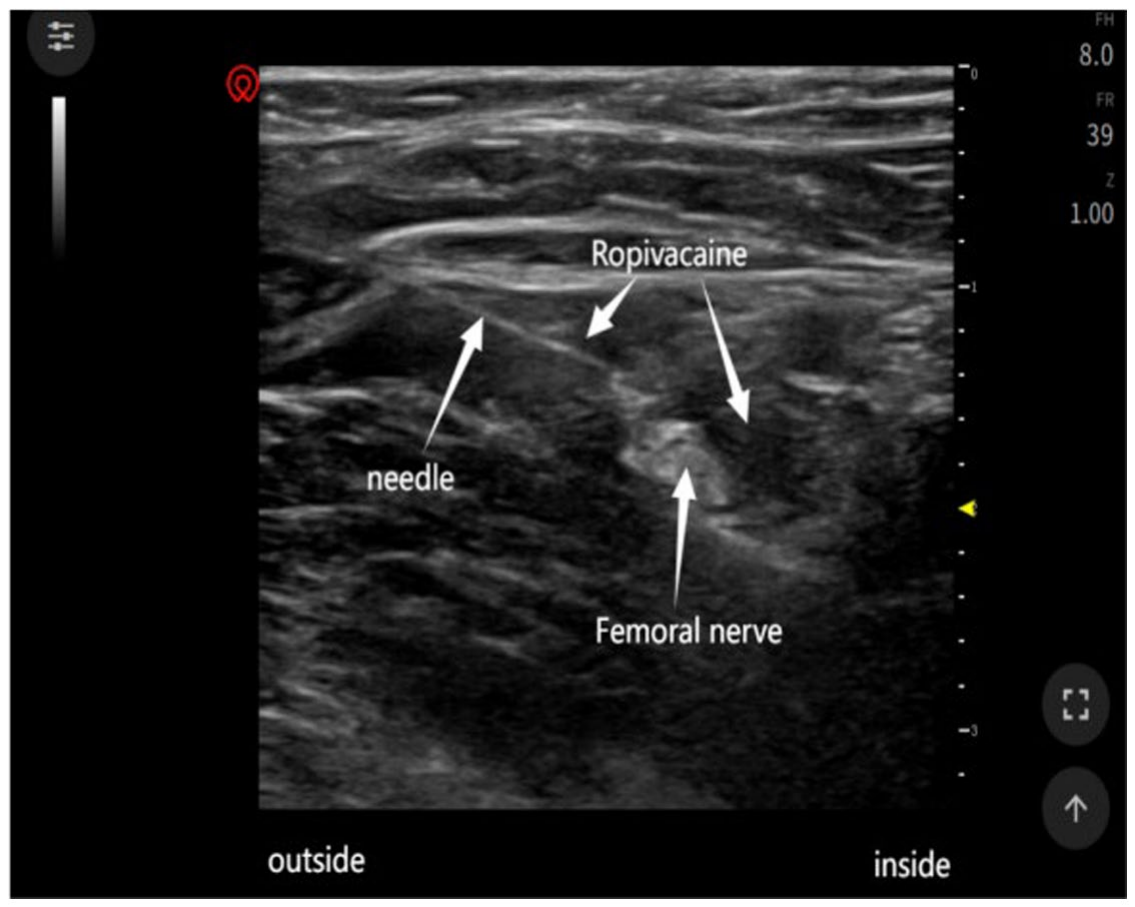
Ultrasound-guided puncture images of the FN.

Upon the patient’s arrival in the room, peripheral venous access was established, and routine monitoring of pulse oximetry, heart rate, five-lead electrocardiogram, and non-invasive blood pressure was initiated. The operating room temperature was maintained at a constant level (22–24 °C) to minimize interference. The FNB was administered by an experienced anesthesiologist specializing in ultrasound-guided regional anesthesia. Prior to the initiation of FNB, patients were administered 1.0–1.5 mg of midazolam for sedation and anxiety management. Oxygen was delivered via a face mask at a rate of 2–4 L/min, and peripheral venous access was established to maintain adequate fluid volume. The patient was positioned supine with the lower limbs in a natural position. A high-frequency ultrasound probe (Huasheng color ultrasound diagnostic system, model: Navi u) was utilized, and with the assistance of color Doppler imaging, the femoral artery and vein were identified. Beneath the probe, the moderately echogenic iliopsoas muscle and the hyperechoic fascia iliaca were observed, covered by the fascia lata. The femoral nerve (FN), located laterally to the femoral artery between the fascia iliaca and the iliopsoas muscle, exhibited a fusiform hyperechoic appearance. Employing an in-plane technique, the puncture was performed from lateral to medial, ensuring the needle tip reached the vicinity of the FN. After confirming no blood return, the medication was administered (0.25% ropivacaine 20 mL). The FN was enveloped and dissociated by the drug solution under real-time ultrasound guidance. Ultrasound imaging of the FN is presented in Figure 1, while ultrasound-guided puncture of the FN is presented in Figure 2.

The PI of the big toe was monitored every 2 mins for a total duration of 20 mins on both the blocked side and the non-blocked side. Simultaneously, the degree of sensory and motor block on the blocked side was assessed at these time points.

(1) PI assessment: Pulse oximetry was conducted using a pulse oximeter (Shenzhen Mindray Biomedical Electronics Co., Ltd., model: Mindray BeneVision N1) to monitor the PI. This compact intelligent transport monitor complies with EN1789, EN13718, several US military standards, and other transportation standards. It ensures reliable data collection and represents the most integrated patient monitor in the industry. To enable real-time, continuous, and uninterrupted monitoring of PI value changes in both limbs, two identical monitors were utilized for data acquisition. Prior to the administration of the FNB, PI values from both monitors were independently compared to confirm the absence of significant inter-device variability. Subsequently, the FNB was performed, followed by continuous PI monitoring throughout the procedure. This approach ensured reliable bilateral PI monitoring while minimizing potential discrepancies between monitoring devices. Two independent devices were placed on the big toes of both lower limbs to measure PI on both the blocked and non-blocked sides. PI values were recorded at baseline (prior to FNB) and at 2-min intervals for 20 min (i.e., PI at 0, 2, 4, 6, 8, 10… 20 min). Throughout the measurement period, patients remained in a consistent environmental setting and position to ensure stable PI readings.
(2) Sensory and motor assessment: The sensory evaluation was conducted using an acupuncture sensation test on the skin innervated by the FN, employing a 24-gauge blunt needle. The results were assessed according to the Hollmen scale, where 0 indicates normal sensation, 1 denotes reduced needling sensation compared to the contralateral limb, 2 signifies perception of the needle as blunt, and 3 represents loss of tactile sensation. For motor function assessment, the grading was as follows: 0 for normal muscle function, 1 for decreased muscle function relative to pre-block status, 2 for significant impairment of muscle motor function, and 3 for complete loss of muscle motor function. Subsequently, irrespective of the success of the FNB, the patient was transferred to the operating room and underwent general anesthesia.
(3) Research Personnel Allocation: A designated physician performed ultrasound-guided FNB. Another independent evaluator, who remained blinded to the intervention, was responsible for assessing the efficacy of the FNB, including PI monitoring and evaluation of sensory and motor block levels. This evaluator did not participate in the nerve block procedure and was unaware of which limb had undergone the intervention. A third physician was assigned to conduct postoperative follow-up.

### Indicators of observation

(1) Main outcome measure: The PI value of the big toe on the blocked side within 20 min. (2) Secondary outcome measures: ① the PI value of the big toe on the non-blocked side within 20 min; ② sensory scores on the blocked side at each time point; ③ motor scores on the blocked side at each time point.

### Statistical analysis

1. Sample size calculation method: The area under the curve (AUC) of the PI value on the blocked side for the subjects served as the outcome measure for observation. Based on the relevant literature (7), an AUC of 0.8 was targeted for detection, with a null hypothesis set at 0.5, a two-sided *α* of 0.05, and a statistical power of 80%. Accounting for the potential failure rate of nerve blocks, the sample size was inflated by 10%. Using PASS 15.0 software, it was determined that a minimum of 78 subjects would be required.
2. Data statistical methods: Statistical analyses were conducted using SPSS 21.0 software. Normally distributed continuous data were presented as mean ± standard deviation (x̄ ± s), and comparisons between groups were performed using one-way analysis of variance (ANOVA). Non-normally distributed continuous data were reported as median (M) with interquartile range (IQR), and group comparisons were conducted using the Mann–Whitney U test. Repeated measures ANOVA with Bonferroni *post-hoc* tests was employed for within-subject comparisons. Categorical data were analyzed using the χ2 test or Fisher’s exact test, as appropriate. A *p*-value < 0.05 was considered statistically significant. To evaluate diagnostic performance, ROC curves were constructed to assess the sensitivity and specificity of PI changes on the block side. The optimal cutoff value was determined based on the ROC curve yielding the maximum Youden index (sensitivity + specificity-1).

## Results

### General information

**Figure 3:**
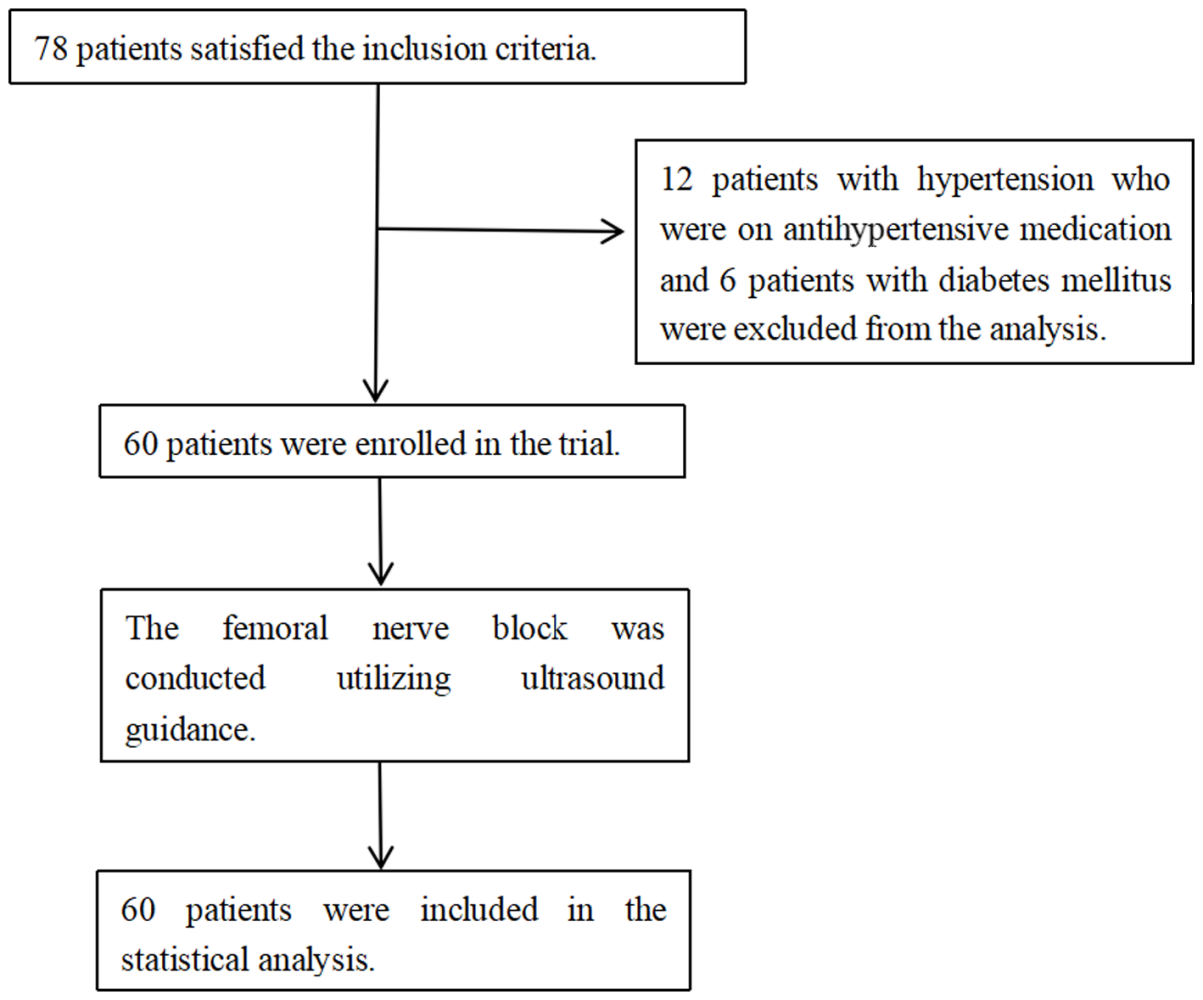
Flow chart of study subjects inclusion.

A total of 78 patients were initially included in this study. Among them, 12 patients with hypertension who were on antihypertensive medication and 6 patients with diabetes mellitus were excluded from the analysis. All patients underwent arthroscopic debridement and meniscus repair or suture of the affected knee. The flow chart for the selection of study subjects is presented in Figure 3. 60 patients were enrolled in the trial, and all underwent successful FNB without any complications related to the procedure or surgery. Ultimately, data from these 60 patients were included in the statistical analysis. The cohort comprised 30 males and 30 females, with a mean age of 33.08 ± 11.49 years and a mean body mass index (BMI) of 23.03 ± 2.69 kg/m^2^. Additionally, 27 patients were classified as ASA grade I and 33 as ASA grade II. Detailed patient demographics are presented in Table 1.

**Table 1:** General data of patients (x ± s).

Indicators	Gender(Male/female) (number of cases)	Age (years)	BMI (kg/m^2^)	ASA classification criteria (I/II) (number of cases)
Results	30/30	33.08 ± 11.49	23.03 ± 2.69	27/33

1 ROC curve analysis of the peripheral PI on both the blocked and non-blocked sides

**Figure 4:**
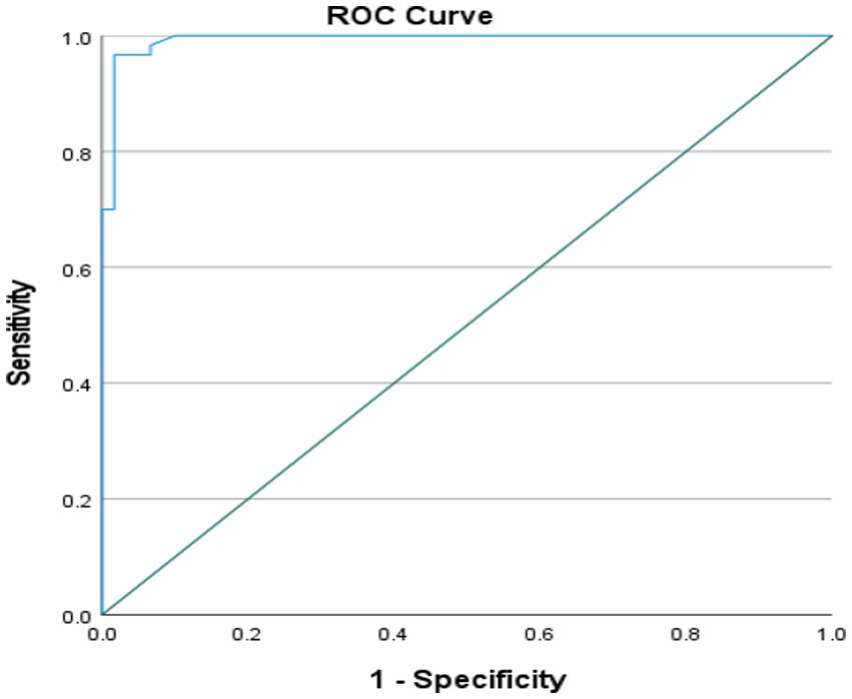
ROC curves of PI values at 6 min post-blockade.

In this study, the ROC curve was constructed, and the area under the ROC curve was calculated to evaluate the predictive capability of PI changes for the success of FNB. Sensitivity and specificity were determined to identify the optimal cut-off value. The results demonstrated that the AUROC of the PI value at the T6 time point on the blocked side was the highest, reaching 0.993 (95% CI: 0.982 to 1.000), with a sensitivity of 96.7%, specificity of 93.3%, Youden index of 0.934, and a cut-off value of 1.755 (as detailed in Table 2; Figure 4). These findings suggest that the change in PI values 6 min post-block holds substantial potential as a predictor of successful FNB.

**Table 2:** Area under the curve.

Test result variable(s)	Area	Std. error^a^	Asymptotic 95% confidence interval	
			Lower bound	Upper bound
PI(T0)	0.542	0.053	0.438	0.645
PI(T2)	0.850	0.035	0.780	0.919
PI(T4)	0.983	0.008	0.966	0.999
PI(T6)	0.993	0.005	0.982	1.000
PI(T8)	0.992	0.005	0.982	1.000
PI(T10)	0.993	0.006	0.981	1.000
PI(T12)	0.993	0.006	0.981	1.000
PI(T14)	0.989	0.008	0.974	1.000
PI(T16)	0.985	0.008	0.969	1.000
PI(T18)	0.980	0.010	0.960	1.000
PI(T20)	0.973	0.012	0.949	0.998

2 Comparison of PI values between the blocked side and the non-blocked side

**Figure 5:**
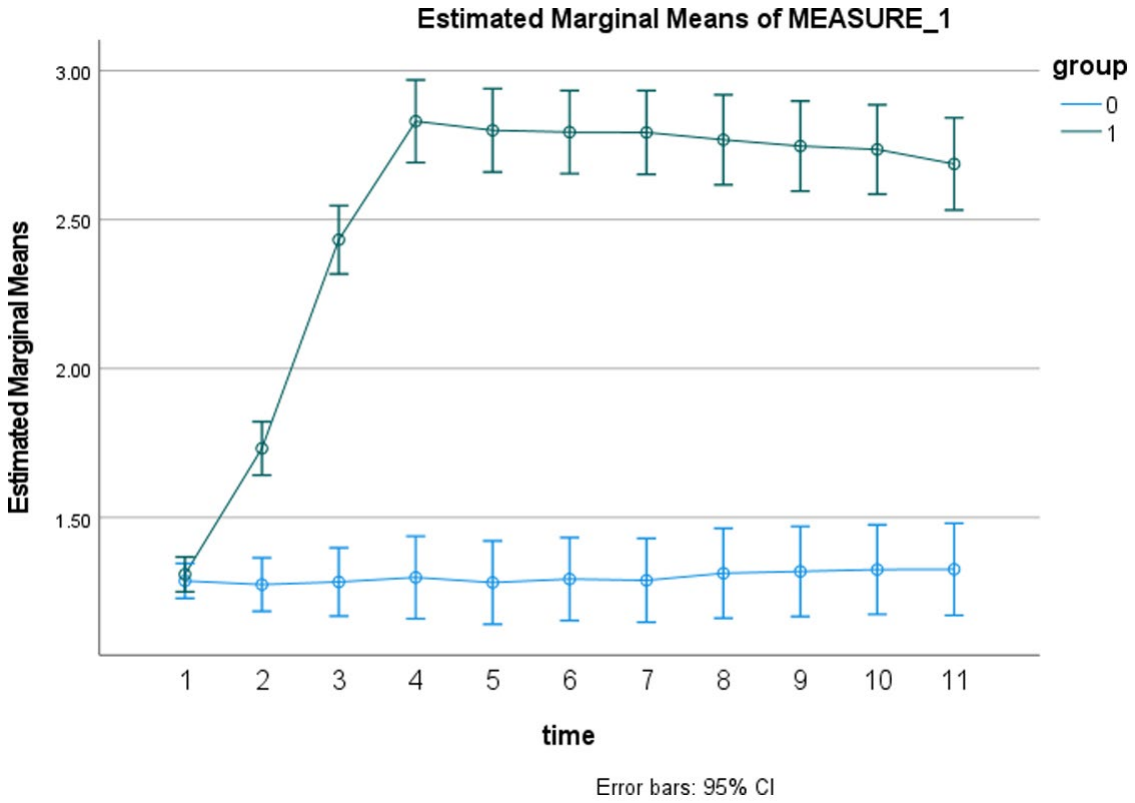
Comparison of PI values at each time point between the blocked side and the non-blocked side. Group (0 = non-blocked side, 1 = blocked side), time (1 = T0, 2 = T2, 3 = T4, 4 = T6, 5 = T8, 6 = T10, 7 = T12, 8 = T14, 9 = T16, 10 = T18, 11 = T20).

Comparisons of PI values between the blocked and non-blocked sides across multiple time points were performed using repeated measures analysis of variance (RM-ANOVA). Mauchly’s test of sphericity indicated a violation of the sphericity assumption (*p* < 0.05); therefore, the Greenhouse–Geisser correction was applied to adjust for this deviation. The analysis revealed a statistically significant difference in PI values over time between the blocked and non-blocked sides (*F* = 140.536, *p* < 0.001). Additionally, significant variations in PI values were observed at different time points on the blocked side (*p* < 0.001), with the most pronounced increase occurring at T6, where the average PI value reached 2.83 ± 0.72, approximately 2.16 times higher than the baseline value. In contrast, no significant changes in PI values were detected on the non-blocked side across all time points (*p* > 0.05). These findings are presented in Figure 5.

3 Comparison of PI values between different genders

**Figure 6:**
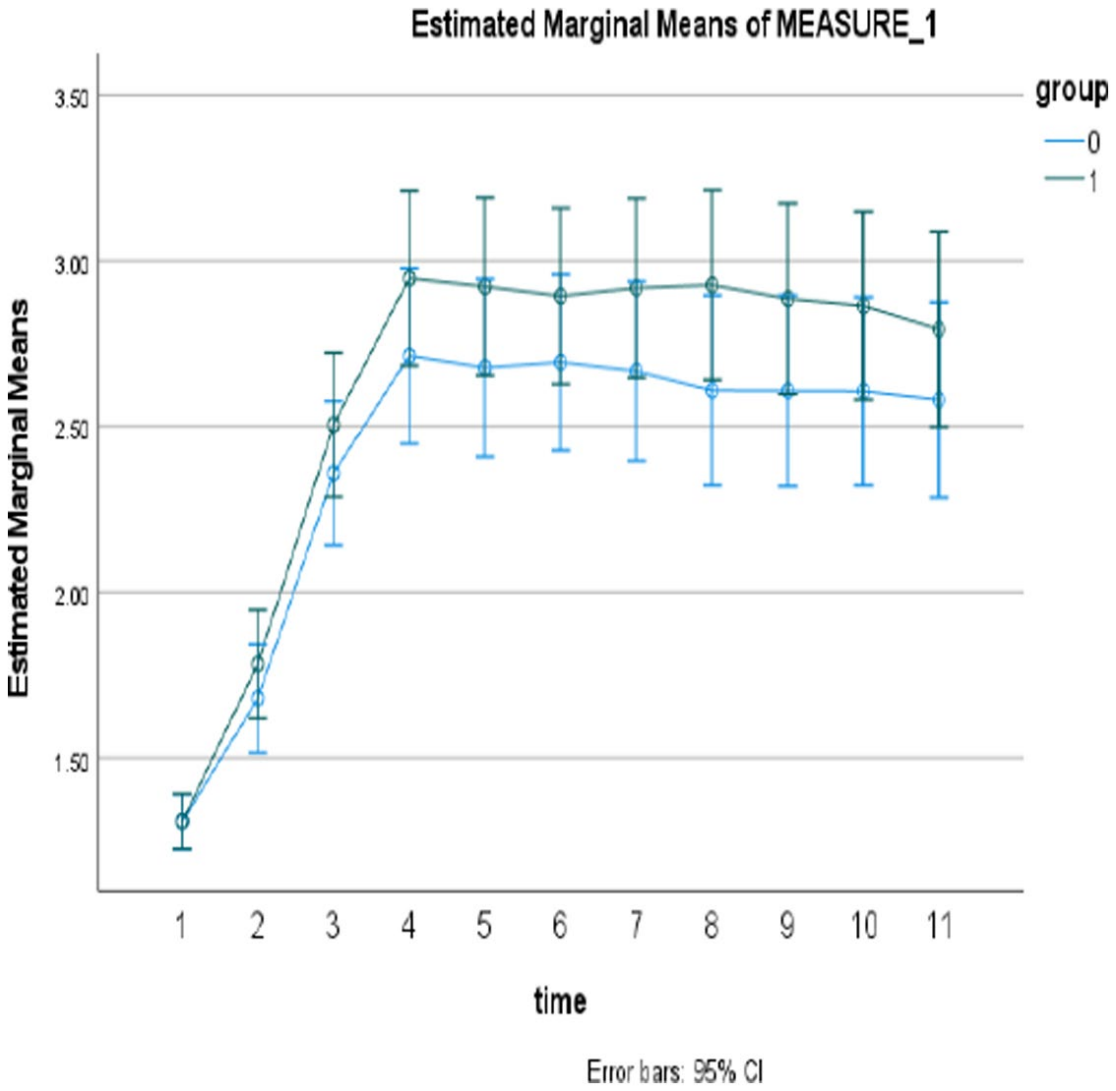
Comparison of PI values between males and females on the blocked side. Group (0 = females, 1 = males), time (1 = T0, 2 = T2, 3 = T4, 4 = T6, 5 = T8, 6 = T10, 7 = T12, 8 = T14, 9 = T16, 10 = T18, 11 = T20).

**Figure 7:**
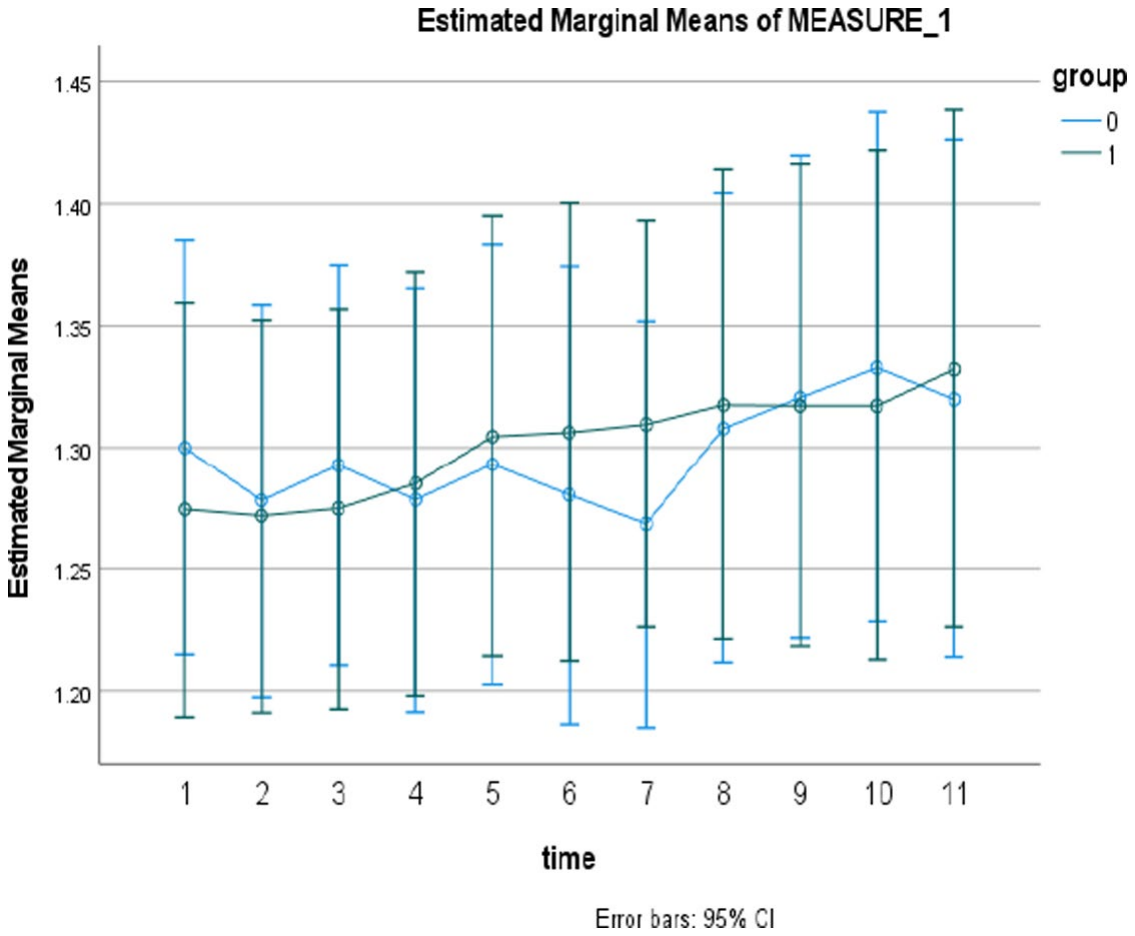
Comparison of PI values between males and females on the non-blocked side. Group (0 = females, 1 = males), time (1 = T0, 2 = T2, 3 = T4, 4 = T6, 5 = T8, 6 = T10, 7 = T12, 8 = T14, 9 = T16, 10 = T18, 11 = T20).

Comparisons of PI values between males and females on the obstructed side across multiple time points were performed using repeated measures analysis of variance (RM-ANOVA). Mauchly’s test of sphericity indicated a violation of the sphericity assumption (*p* < 0.05); therefore, the Greenhouse–Geisser correction was applied to adjust for this deviation. The analysis revealed a statistically significant difference in PI values over time between males and females on the obstructed side (*F* = 145.413, *p* < 0.001), as presented in Figure 6. In contrast, no statistically significant difference in PI values was observed between males and females on the non-blocked side across time points (*F* = 2.760, *p* > 0.05), as presented in Figure 7.

4 Evaluation of the extent of sensory blockade on the affected side

According to the Hollmen scale, a score of 3 indicates a complete block. The results demonstrated that none of the patients achieved a complete block 6 min post-block. One patient (1.67%) exhibited a complete block 8 min post-block, four patients (6.67%) at 10 min, and 33 patients (55%) at 16 mins. A total of 58 patients (96.7%) achieved a complete block by 20 min post-block, as detailed in Table 3.

**Table 3:** Comparison of complete sensory and motor block on the affected side in patients.

Time (minutes)	Sensory block (number of cases/percentage)	Motor block (number of cases/percentage)
0	0,0	0,0
2	0,0	0,0
4	0,0	0,0
6	0,0	0,0
8	1,1.67%	0,0
10	4,6.67%	2,3.3%
12	7,11.67%	3,5%
14	17,28.3%	5,8.3%
16	33,55%	11,18.3%
18	53,88.3%	23,38.3%
20	58,96.7%	43,71.6%

5 Evaluation of the extent of motor blockade on the affected side

Based on the degree of motor block, a score of 3 indicates a complete block. The results indicated that no patients achieved a complete block at 6 min post-block. At 10 min, 2 patients (3.3%) exhibited a complete block; this increased to 3 patients (5%) at 12 min and 28 patients (38.3%) at 18 min. By 20 min post-block, 43 patients (71.6%) had achieved a complete block. These findings are summarized in Table 3.

6 Comparison of sensory and motor block scores at different time points on the blocked side

**Figure 8:**
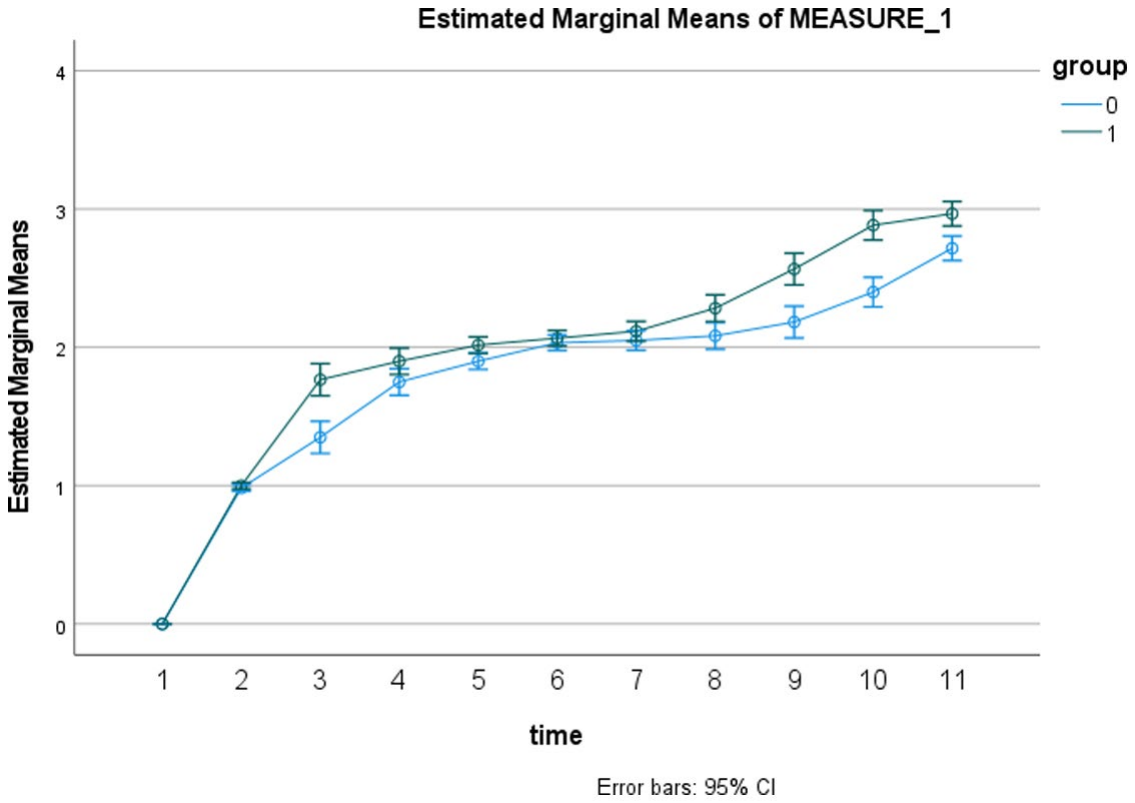
Comparison of sensory and motor block scores at different time points on the blocked side. Group (0 = motor block score, 1 = sensory block score), time (1 = T0, 2 = T2, 3 = T4, 4 = T6, 5 = T8, 6 = T10, 7 = T12, 8 = T14, 9 = T16, 10 = T18, 11 = T20).

Comparisons of sensory and motor block scores on the blocked side across multiple time points were performed using repeated measures analysis of variance (RM-ANOVA). Mauchly’s test of sphericity indicated a violation of the sphericity assumption (*p* < 0.05); therefore, the Greenhouse–Geisser correction was applied to adjust for this deviation. The analysis revealed a statistically significant increase in sensory block scores over time on the blocked side (*F* = 840.816, *p* < 0.001), as presented in Figure 8.

## Discussion

The current study demonstrated that the PI values on the blocked side increased rapidly at 6 min post-FNB, whereas the PI values on the non-blocked side remained relatively stable without significant changes. Specifically, the mean PI value at T6 on the blocked side was 2.83 ± 0.72, representing a 2.16-fold increase compared to the baseline value. The AUROC was 0.993 (95% CI: 0.982 to 1.000), with a sensitivity of 96.7%, specificity of 93.3%, Youden index of 0.934, and a cut-off value of 1.755. Notably, this study observed that the increase in PI values on the blocked side was more pronounced in male patients compared to female patients, with a statistically significant difference (*p* < 0.001). The change in PI value of the big toe measured 6 min after the block demonstrated considerable potential as a predictive indicator for the success of FNB.

The blood supply to the hallux of the lower limb originates from the continuation of the femoral artery and vein, with the FN in close proximity to these vessels. Ultrasound imaging technology can clearly delineate the FN and its surrounding tissue structures, guiding the needle accurately to the injection target and enabling real-time dynamic observation of the diffusion of local anesthetics around the FN (8). The sequence of block for local anesthetics on nerve fibers typically progresses from distal to proximal. Initially, autonomic nerve fibers are affected, resulting in sympathetic attenuation and/or parasympathetic dysfunction. Subsequently, sensory nerve fibers are blocked, followed by motor nerve fibers, ultimately leading to motor paralysis and loss of pressure sensation (9). Following FNB, sympathetic nerve block is initially induced, leading to a reduction in local vascular tone and an increase in local blood flow (5). Consequently, a rapid increase in the PI value of the hallux was observed shortly after the block, while no significant changes were noted in the PI on the non-blocked side.

The primary determinants of the PI include peripheral vascular tone and stroke volume. Any variables that alter these physiological parameters may influence PI measurements. Such variables encompass demographic and clinical factors such as age, gender, and body position (8). Nishimura et al. (10) examined the relationship between the change in PI and demographic factors such as gender and age. A total of 70 healthy volunteers were recruited and categorized into four groups: young men, young women, older men, and older women. The results indicated that the PI was significantly lower in young men compared to older men (*p* = 0.0039) and older women (*p* = 0.0095). Furthermore, the PI value in young women was also significantly lower than that in older men (*p* = 0.0011) and older women (*p* = 0.0028). Another study (11) examined the impact of postural changes on physiological indices. 40 healthy volunteers were randomly allocated into four groups based on their positions: supine (Group S), Trendelenburg (Group T), reverse Trendelenburg (Group R), and prone (Group P). The findings revealed that the PI in Group T was significantly higher than in Group S (*p* < 0.05). Conversely, the PI in Group R was significantly lower than in Group S (*p* < 0.05). No significant difference was observed between Group P and Group S (*p* > 0.05). Elderly patients exhibit various physiological and pathological alterations that may affect clinical monitoring parameters. These include diminished cardiac reserve capacity and reduced stroke volume (12); age-related structural and functional changes in peripheral blood vessels, resulting in increased vascular resistance and endothelial dysfunction (13, 14); as well as decreased neural excitability, impaired motor and sensory function, and compromised autonomic nervous system activity (15). Collectively, these factors may contribute to potential inaccuracies in PI monitoring. This study enrolled a cohort of young and middle-aged patients with a balanced male-to-female ratio. All procedures were conducted in the supine position, with controlled variables that could potentially influence the PI, thereby enhancing the rigor of the study design. Prior to performing FNB, we ensured optimal room temperature control, provided patients with oxygen inhalation, midazolam sedation, and fluid infusion to alleviate anxiety and prevent complications arising from hypovolemia. All FNBs were successfully administered without any associated complications.

The present study demonstrated that the PI value increased rapidly 6 min following FNB, with a cutoff value of 1.755 on the blocked side. This cutoff value is comparable to that reported by Galvin et al. (16), who identified a successful cutoff value of 1.55 for axillary brachial plexus block in upper limb surgery. Wu Xinhai et al. (17) compared the intermuscular sulcus and axillary brachial plexus block approaches, finding that the PI of the intermuscular sulcus approach increased 6 min post-block, whereas the axillary approach showed an increase 8 min post-block, relative to the non-blocked limb. Sebastiani et al. (18) utilized the difference in PI between both limbs to evaluate the effectiveness of the interscalene brachial plexus block, demonstrating a significant increase in the PI difference between sides 5 min after successful block. These findings align with our study, where PI values in the blocked limb were notably elevated several minutes following the block. In their study of inclavian brachial plexus block, Bereket et al. (19) reported that the mean PI value measured 10 min post-block was 6.4 ± 2.3, with a cutoff value greater than 9.2. Similarly, Abdelnasser et al. (7) demonstrated a significant difference in PI values 10 min after the block. However, given that the observation time points in their study were set at 0, 10, 20, and 30 min, the relatively large intervals between these points may introduce certain limitations to the results. In this study, the monitoring interval was reduced to 2 mins, allowing for more frequent assessment of PI changes. Additionally, the degree of sensory and motor block was evaluated to determine the optimal time point.

Notably, this study observed that the increase in PI values on the blocked side was more pronounced in male patients compared to female patients, with a statistically significant difference (*p* < 0.001). The PI serves as an indicator of the degree of peripheral vasodilation following nerve block. When administered the same dose of the same local anesthetic, males exhibited greater vasodilatory responses, as reflected by a more substantial elevation in PI values. In contrast, no statistically significant difference in PI values was observed on the non-blocked side between genders, which may be attributed to variations in vascular reactivity, endothelial function, and pharmacokinetic profiles between men and women. A recent prospective study (20) explored the impact of biological sex on the pharmacodynamic, pharmacokinetic, and morphometric characteristics of peripheral nerve block. The findings revealed that the pharmacodynamic properties of peripheral regional blockade were not influenced by biological sex, whereas pharmacokinetic parameters demonstrated gender-related variations. Specifically, women may require higher drug doses to achieve comparable block efficacy. Additionally, a multicenter study (21) reported significant differences in pulse oximetry parameters among healthy adult males and females; however, the underlying causes for these discrepancies were not investigated in depth.

In the traditional evaluation method, we utilized the Hollmen scale to assess the degree of sensory block through acupuncture sensation testing. At 16 min post-block, only 55% of patients achieved complete sensory block, whereas at 20 min post-block, this proportion increased to 96.7%. However, in terms of motor block assessment, only 71.6% of patients demonstrated complete block at 20 min post-block. The findings of this study are in agreement with those reported by Lal et al. (22), who also examined sensory and motor block onset in patients undergoing supraclavicular brachial plexus block. Their results indicated that sensory block initiation occurred at 5 min, while motor block onset was observed at 10 min. Complete sensory and motor blocks were achieved at 15 and 20 min, respectively.

The administration of local anesthetics initially blocks the sympathetic nerves, followed by the sensory nerves, and ultimately the motor nerves. Additionally, the efficacy of nerve block is influenced by the type, concentration, and dosage of the local anesthetic employed. Yin Yulin et al. (23) demonstrated in their study on ultrasound-guided brachial plexus block combined with nerve stimulation that the onset times of both motor and sensory blocks were significantly shorter in the medium concentration group (0.375% ropivacaine) and high concentration group (0.5% ropivacaine) compared to the low concentration group (0.25% ropivacaine). Additionally, the duration of both motor and sensory blocks was significantly longer in the medium and high concentration groups than in the low concentration group (*p* < 0.05). However, the incidence of adverse reactions was also found to be higher in the high concentration group. In this study, general anesthesia in combination with FNB was employed, utilizing a low concentration of ropivacaine. This approach not only ensured effective analgesia and minimized adverse reactions but also facilitated early postoperative rehabilitation training for patients, aligning with the principles of ERAS.

The evaluation of nerve block efficacy in non-cooperative patients, such as pediatric individuals and those with psychiatric comorbidities, remains a major challenge in clinical practice. Due to impaired communication or lack of cooperation, these patients are at increased risk of technical difficulties during nerve block procedures, including inaccurate needle placement, incomplete blockade, and potential injury to nerves or blood vessels—factors that collectively elevate the likelihood of block failure. Consequently, nerve block procedures are often performed under general anesthesia, which complicates the real-time assessment of block effectiveness. In such scenarios, anesthetic management is typically adjusted based on intraoperative noxious stimuli rather than direct feedback from the patient. The utility of PI monitoring offers a promising solution to this clinical dilemma. A study conducted in pediatric patients (24) demonstrated that epidural block administered under sedation and analgesic anesthesia resulted in a marked decrease in PI following drug administration. This decline exhibited 100% sensitivity and specificity, suggesting that a 30% reduction in PI may serve as a reliable early indicator for detecting intravascular injection of the epidural test dose.

The PI serves as an objective monitoring parameter, with data automatically acquired by the monitoring device, thereby eliminating reliance on subjective patient feedback. It provides continuous and real-time visualization of peripheral perfusion changes, enabling prompt assessment of the nerve block effect. PI monitoring is highly sensitive and capable of predicting the success or failure of nerve blockade within minutes. This significantly reduces the time required for evaluation, expedites surgical turnover, and allows anesthesiologists to promptly modify the anesthetic strategy. As a result, the efficiency of knee arthroscopy procedures is enhanced, aligning well with the principles of ERAS.

This study also has certain limitations that warrant acknowledgment. First, it is a single-center investigation with a relatively small sample size; thus, multi-center studies with larger samples could be conducted in the future to further validate these findings. Second, this study was limited to healthy young patients and did not investigate the potential influence of population diversity, age variation, or gender differences on PI. Future research should address these variables to generate more comprehensive clinical evidence for broader patient populations.

## Conclusion

The PI is an effective predictor of FNB success. A PI value above 1.755 indicates a successful block, whereas minimal change suggests failure. This enables anesthesiologists to promptly adjust anesthesia protocols, thereby improving the efficiency of knee arthroscopy and aligning with ERAS principles.

## Data Availability

The raw data supporting the conclusions of this article are available upon reasonable request to the corresponding author.
